# Xerostomia in the menopausal transition: are we measuring menopause or its comorbidities?

**DOI:** 10.1016/j.clinsp.2026.101154

**Published:** 2026-10-01

**Authors:** Giovanna P. Florezi, Lara Vanelli, Fernanda Pereira de Moraes, Isabel Cristina Esposito Sorpreso, José Maria Soares-Junior, Silvia Vanessa Lourenço

**Affiliations:** a Surgery Department, Faculdade de Medicina, Universidade de São Paulo, São Paulo, SP, Brazil; b LIM-06, Instituto de Medicina Tropical, Faculdade de Medicina, Universidade de São Paulo, São Paulo, SP, Brazil; c Obstetrics and Gynecology Department, Faculdade de Medicina da Universidade de São Paulo, São Paulo, SP, Brazil; d Instituto de Estudos Avançados, Universidade de São Paulo, São Paulo, SP, Brazil

## Abstract

• Most studies of salivary flow in menopause exclude comorbidities and medication use.
• This removes the very women in whom dry mouth is most common in clinical practice.
• When measured, medication and comorbidity predict xerostomia better than menopause.
• We propose criteria to measure and adjust for confounders rather than exclude them.

Xerostomia has been considered as a hallmark of the menopausal transition and post-menopause; it has been classically established as a consequence of hypoestrogenism.^1^ This association, however, is supported on a body of evidence that appears to be characterized by methodological shortcomings and conflicting findings. Our research group has set up the first Observatory on Oral Heath in Menopause, at the Hospital das Clínicas, Universidade de São Paulo; our clinical practice reveals that the complaint of dry mouth in menopausal women is part of a myriad of morbidities that are installed along the period of menopausal transition and post-menopause, the Menopausal Associated Metabolic Syndrome (MAMS). Patients present multiple systemic comorbidities and use varied medications, which may interfere with salivation and enhance xerostomia symptoms.

The aim of this communication is to highlight the strategic importance of the oral cavity as an integral indicator of women's systemic health during the menopausal transition, and post-menopause within the broader agenda of the One Health concept. We advocate that advancing this field will require not only a shift in conceptual thinking but also the adoption of rigorous study designs capable of accurately disentangling the biological effects of hypoestrogenism from the influence of aging, multimorbidity, lifestyle, and polypharmacy. Only through methodologically rigorous, clinically representative, and multidisciplinary research will it be possible to generate the robust evidence needed to establish the oral cavity as a sentinel of women's systemic health during the menopausal transition, thereby advancing precision medicine within the One Health framework.

Analysis of the available studies on salivary flow in peri- and postmenopausal women suggests that the inclusion and exclusion criteria adopted across investigations often reflect study design convenience rather than a biologically grounded assessment of the effects of hypoestrogenism on salivary gland function. We argue that meaningful progress in this field would depend less on conducting additional studies that replicate the same methodological approach and more on establishing standardized criteria that measure and appropriately adjust for the burden of comorbidities and the use of xerogenic medications, rather than simply excluding these clinically relevant factors.

When studies objectively assessing salivary flow in peri- and postmenopausal women are critically examined, two opposing methodological approaches emerge, neither of which allows xerostomia to be confidently attributed only to menopause itself. The first, and most prevalent, approach seeks to “purify” the study population by excluding women with systemic diseases or those taking medications in an attempt to isolate the hormonal effects of estrogen deficiency.^2^, ^3^, ^4^, ^5^, ^6^, ^7^, ^8^ For example, Rukmini et al. excluded participants with diabetes, hypertension, or any medication use,^5^ while Agha-Hosseini et al. additionally excluded smokers and women with obesity.^2^ Although this strategy reduces potential confounding, it also systematically removes the population in whom xerostomia is most commonly observed in clinical practice: middle-aged women frequently affected by hypertension, diabetes, thyroid disorders, depression, and polypharmacy. As a result, the findings may have limited external validity, provide only restricted insight into the real-world contribution of menopause to salivary gland dysfunction and hamper the validation of oral cavity as an indicator of health.

More importantly, even after applying these highly restrictive eligibility criteria, the putative independent effect of menopause on salivary flow remained inconsistent. Agha-Hosseini et al.^2^ found no significant difference in stimulated salivary flow between women with and without xerostomia (1.38 vs. 1.37 mL/min; p = 0.837),^2^ while Divya Harika et al. likewise reported comparable salivary flow rates in pre- and postmenopausal women.^7^ In the few studies reporting reduced salivary flow in postmenopausal women,^4^^,^^6^ the comparison groups consisted of substantially younger, reproductive-age women, making it difficult to disentangle the effects of menopause from those of chronological aging.

In contrast, the second methodological approach, although less frequently adopted, provides substantially stronger evidence because it preserves the clinical complexity inherent to the menopausal population while explicitly quantifying the contribution of potential confounders. Rather than eliminating women with multimorbidity and medication use, these studies incorporated these variables into the analytical framework and consistently demonstrated that comorbidities and xerogenic medications are more robust predictors of xerostomia than menopausal status per se. Ben-Aryeh et al.,^1^ after stratifying participants according to the presence of systemic disease and medication use, observed no significant differences in salivary flow or salivary composition between groups. Likewise, Shinohara et al.^9^ identified the number of medications and the use of xerogenic and psychotropic drugs as the strongest independent determinants of xerostomia. Krupa et al.^10^ further demonstrated that the apparent association between menopausal duration and salivary flow disappeared after adjustment for age, medical history, and medication use, indicating that the crude association was largely explained by these covariates. Consistent with these findings, García-Alfaro et al.,^11^ in a cohort of approximately 3,000 women, identified xerogenic medication use as one of the strongest predictors of xerostomia, with symptom severity increasing in parallel with the number of prescribed medications. Collectively, these studies suggest that the clinical burden traditionally attributed to menopause may be more accurately explained by the accumulation of age-related multimorbidity and polypharmacy than by hypoestrogenism alone.

Beyond these contrasting methodological strategies, the available evidence is further constrained by a series of pervasive limitations that collectively undermine the strength and interpretability of the literature. Most studies rely on small convenience samples recruited from dental or gynecological clinics, limiting representativeness and external validity. Comparisons across studies are further hindered by substantial heterogeneity in sialometric protocols, including the assessment of unstimulated versus stimulated saliva, the use of different stimulatory agents, and variations in collection techniques. Additional inconsistency arises from the lack of standardized definitions of menopausal status, with peri- and postmenopausal women frequently analyzed as a single group despite their distinct endocrine profiles. Finally, many studies fail to distinguish xerostomia, a subjective symptom, from hyposalivation, an objective physiological measurement, often treating the two entities as interchangeable despite compelling evidence that xerogenic medications may induce xerostomia independently of salivary flow.^12^ Taken together, these methodological shortcomings substantially limit causal inference and contribute to the persistent uncertainty regarding the independent role of menopause in salivary gland dysfunction.

Taken together, these findings suggest that the reported burden of xerostomia attributed to menopause has likely been distorted by selection bias and insufficient control of multimorbidity and medication-related confounding. Consequently, the independent contribution of hypoestrogenism to salivary gland dysfunction remains uncertain. Addressing this question will require adequately powered, population-based longitudinal cohorts that capture the clinical complexity of menopausal women, rather than highly selected populations, and integrate comprehensive salivary phenotyping with advanced molecular approaches, including transcriptomics, proteomics, metabolomics, and other multi-omics technologies. Although such studies inevitably demand substantial financial investment, their potential to generate robust mechanistic evidence and clinically actionable biomarkers far outweighs the associated costs. By clarifying the biological and clinical determinants of xerostomia in menopause, this next generation of studies could redefine the understanding of women's oral health during midlife and establish new opportunities for precision prevention, early diagnosis, and targeted therapeutic interventions.

## Data availability statement

The datasets generated and/or analyzed during the current study are available from the corresponding author upon reasonable request.

## Conflicts of interest

The authors declare no conflicts of interest.
